# A neonicotinoid pesticide alters *Drosophila* olfactory processing

**DOI:** 10.1038/s41598-023-37589-w

**Published:** 2023-06-30

**Authors:** Anna R. Tatarko, Anne S. Leonard, Dennis Mathew

**Affiliations:** Department of Biology, University of Nevada-Reno, Reno, NV 89557 USA

**Keywords:** Neuroscience, Olfactory system, Sensory processing, Agroecology

## Abstract

Neonicotinoid pesticides are well-known for their sublethal effects on insect behavior and physiology. Recent work suggests neonicotinoids can impair insect olfactory processing, with potential downstream effects on behavior and possibly survival. However, it is unclear whether impairment occurs during peripheral olfactory detection, during information processing in central brain regions, or in both contexts. We used *Drosophila melanogaster* to explore the potential for neonicotinoids to disrupt olfaction by conducting electrophysiological analyses of single neurons and whole antennae of flies exposed to varying concentrations of the neonicotinoid imidacloprid (IMD) that were shown to cause relative differences in fly survival. Our results demonstrated that IMD exposure significantly reduced the activity of a single focal olfactory neuron and delayed the return to baseline activity of the whole antenna. To determine if IMD also impacts olfactory-guided behavior, we compared flies’ relative preference for odor sources varying in ethanol content. Flies exposed to IMD had a greater relative preference for ethanol-laced pineapple juice than control flies, demonstrating that neuronal shifts induced by IMD that we observed are associated with changes in relative preference. Given the interest in the sensory impacts of agrochemical exposure on wild insect behavior and physiology, we highlight the potential of *Drosophila* as a tractable model for investigating the effects of pesticides at scales ranging from single-neuron physiology to olfactory-guided behavior.

## Introduction

Insects are experiencing alarming declines in abundance around the globe^1,2^. These declines have gained attention for the threat they pose to ecosystem services, including pollination, nutrient cycling, and food web stability^1–3^. Principal among the factors implicated in these declines are human-induced stressors such as habitat loss, climate change, invasive species, and agricultural intensification^4^. For example, insecticides, fungicides, and herbicides have become ubiquitous in agricultural settings and urban environments (including backyard gardens) and can even be detected in conservation areas in a broad array of plant taxa^5–7^. Although these chemicals control pests and plant pathogens, non-target insects such as bees and butterflies encounter them while foraging from chemical-laden plants. These chemicals can also have non-consumptive effects, such as creating odor pollution, which makes olfactory-based tasks such as foraging and navigation challenging for insects^8,9^. However, once consumed, they have detrimental impacts on non-target insect health, which can persist for generations after exposure^10–14^.

Neonicotinoids have quickly grown to become the most widely used insecticide in the world^15^. This class of pesticide has received considerable attention for its effects on non-target insects such as pollinators^16–19^. These chemicals are structurally similar to nicotine and have many sublethal effects on insect biology, including but not limited to locomotion, reproduction and growth, behavior and learning, immune function, and nervous system performance^20–22^. A well-documented effect of neonicotinoids on insect nervous systems relates to their influence on sensory performance, particularly olfactory performance^11,23^. For example, Muth et al. found that a sublethal dose of a neonicotinoid impaired bumble bees’ (*Bombus impatiens*) ability to distinguish odors but not colors, indicating that neonicotinoids may have differential effects on sensory systems^11^.

Neonicotinoids act on nicotinic acetylcholine receptors (nAChRs), which transmit olfactory information throughout the insect nervous system^24^ and likely cause disruptions to both peripheral and central olfactory processing. For example, neonicotinoid exposure can affect neurons in the peripheral antennal lobes^25,26^, while more centrally, neonicotinoid exposure can inhibit Kenyon cell activity in the mushroom bodies^27^. Neonicotinoids might also affect the activity of olfactory sensory neurons (OSN), impacting the transmission of olfactory information and affecting its quality before more central processing can occur. Although a 2014 study by Rabhi et al.^26^ did not find changes in OSN response to a pheromone blend in moths exposed to the neonicotinoid Clothianidin, it is unclear if these results generalize across taxa, pesticides, and olfactory contexts.

We aimed to identify the peripheral effects of a neonicotinoid pesticide on olfactory processing using *Drosophila melanogaster.* This model’s well-characterized olfactory physiology allowed us to assess potential impacts scaling from the activity of single neurons, to whole antennal function, to individual fly behavior across three separate experiments. First, we confirmed our protocol of *D. melanogaster* exposure to the neonicotinoid imidacloprid (IMD) by performing a survival assay (Exp. 1). Then, at the single neuron level, we assessed whether IMD alters the sensitivity of a single OSN to a focal odor (Exp. 2). Next, we tested whether IMD alters sensitivity to a focal odor at the whole antenna level (Exp. 3). Finally, we asked if IMD affects performance in an olfactory preference assay (Exp. 4). *Drosophila melanogaster* is a powerful system to explore the effects of pesticide exposure on sensory processing, as OSN structure and function are well-conserved across insects, including beneficial pollinator taxa such as bees and butterflies, where we have fewer tools to identify the potential mechanisms by which pesticide exposure may affect sensory systems^24,28^.

## Results

### Experiment 1: *Drosophila melanogaster* survival is affected by IMD exposure

**Figure 1:**
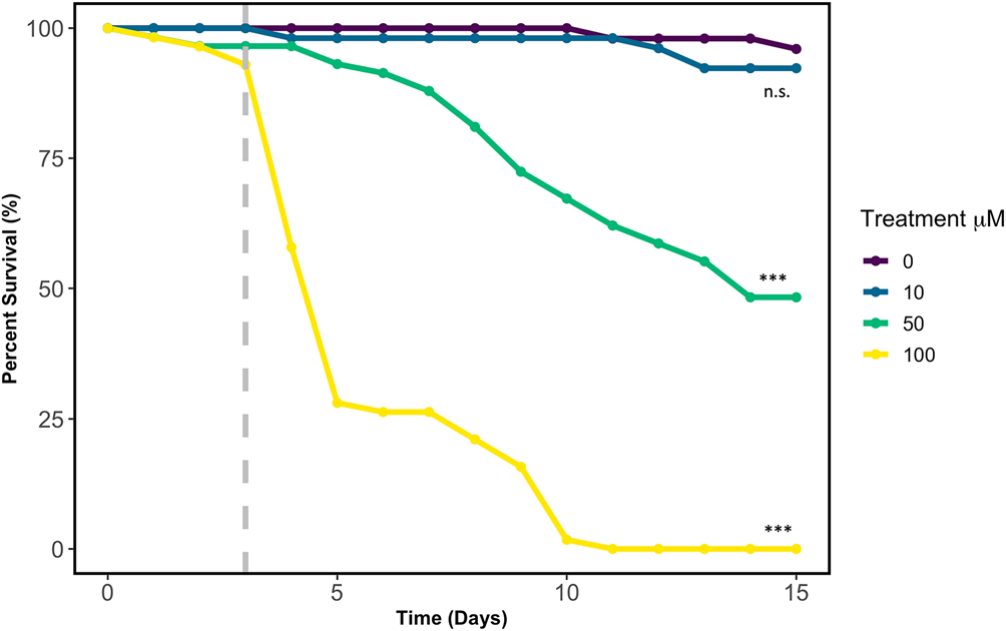
Percent survival of *D. melanogaster* over time under each of the four imidacloprid treatments (0, 10, 50, and 100 µM). ***P < 0.0001, *n.s.* not significant. Gray dashed line at day 3 indicates the age of the flies used in subsequent experiments.

To confirm our exposure protocol of IMD for *D. melanogaster* following^29^, we transferred newly eclosed adult flies (WT Canton-S) to media (see “Methods”) containing 0, 10, 50, or 100 µM of IMD (*N* = 50, 53, 58, and 57 respectively) and observed fly survival across 15 days. We performed pairwise comparisons of survival using a Log-Rank test to assess survival across treatments. We found no difference in survival between control flies and flies reared on a 10 µM diet (Fig. 1, log-rank pairwise comparison, *P* = 0.44). However, 50 and 100 µM diets significantly reduced survival relative to the control (log-rank pairwise comparison, *P* < 0.001 for each pairwise combination).

### Experiment 2: IMD exposure causes concentration-dependent effects on single neuron function

**Figure 2:**
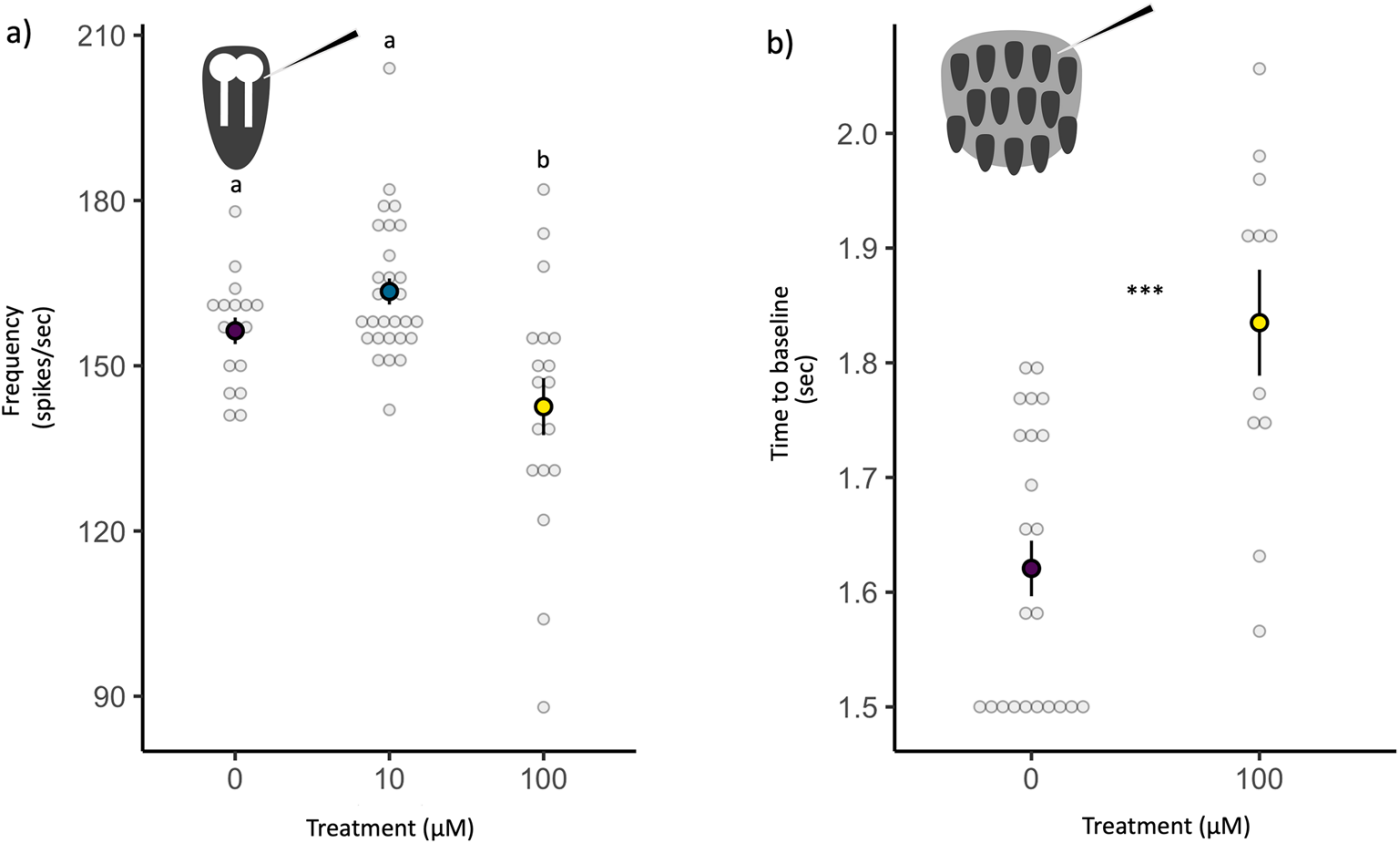
The olfactory sensory neuron (OSN) response to an odor among flies maintained on three concentrations of imidacloprid. (**a**) The mean spikes per second by treatment are noted with bolded dots, ± standard error with the raw recording spike frequency in gray circles. Letters denote statistically distinct groups as identified by a Tukey post-hoc comparison. The top left corner of the figure includes a diagram of how electrophysiology responses were recorded from a sensillum, with OSN shown in white. (**b**) Time of electroantennogram to return to baseline activity after odor stimulation, grouped by treatment. The mean is shown with a bold dot with ± standard error, and each recording’s time to return to baseline is shown in gray circles. Asterisks denote statistically significant differences by treatment (***P < 0.001). The top left corner of the figure includes a diagram of how electroantennogram responses were recorded from the whole antenna, with the sensillum shown in dark gray.

To understand the potential impacts of IMD exposure on individual olfactory sensory neuron (OSN) activity, we performed single-unit recordings on flies (*N* = 14) after 3 days of IMD exposure. Flies were either dosed with either a control (0 µM), a low dose of IMD which Exp. 1 showed caused no difference in fly survival relative to the control (10 µM), or the concentration which caused the most extreme difference in fly survival (100 µM; see Exp 1). We then measured the activity of single neurons (Fig. S1a). We found that IMD treatment significantly affected OSN activity in a concentration-dependent manner (Fig. 2a, *X*^2^ = 18.06, *P* < 0.001). Although there was no difference in OSN activity between control flies and those maintained on a 10 µM IMD diet (post-hoc Tukey HSD tests: *P* = 0.44), flies reared on the 100 µM IMD diet had significantly lower OSN activity compared to the 0 and 10 µM IMD treatments (post-hoc Tukey HSD tests: *P* = 0.002 and < 0.001, respectively).

### Experiment 3: IMD exposure delays return to baseline antennal activity

Focusing on the treatment where we observed an effect in Exp. 2, we compared the whole antennal responses (electroantennograms, EAGs) of flies (*N* = 9) exposed to 0 vs. 100 µM IMD (Fig. S1b). We found no differences between treatments in either the minimum response or the response integral (minimum response: *X*^2^ = 0.932, *P* = 0.334; response integral: *X*^2^ = 2.44, *P* = 0.12). However, the antennae of flies exposed to 100 µM of IMD took longer than control flies to return to baseline activity after odor stimulation (Fig. 2b, *X*^2^ = 24.67, *P* < 0.001).

### Experiment 4: IMD exposure altered performance in an olfactory test

**Figure 3:**
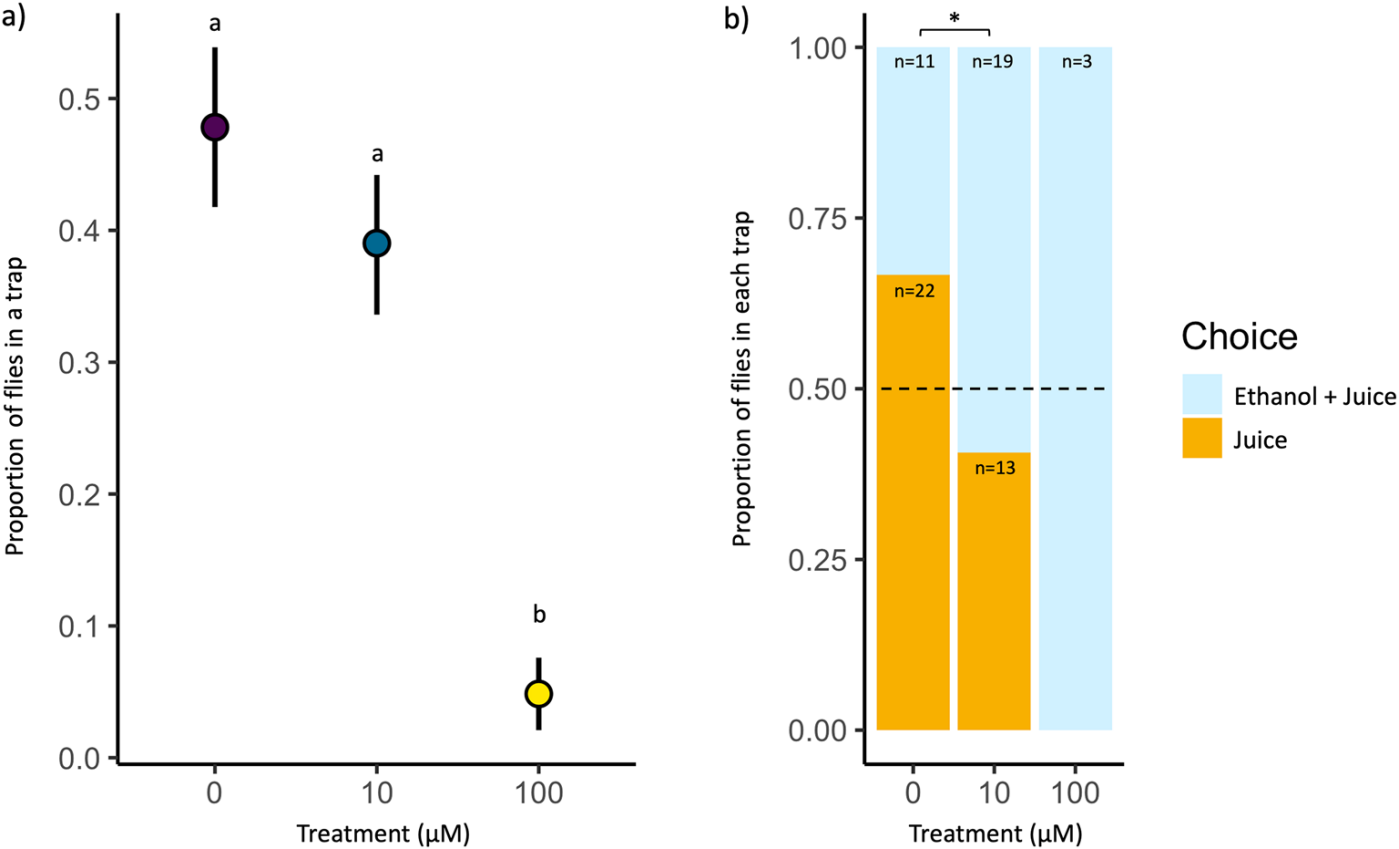
(**a**) The proportion of flies (out of all tested) found in either trap by treatment. The mean is plotted in color as a bold dot with ± standard error. Letters denote statistically distinct groups as identified by a Tukey post-hoc comparison. (**b**) The proportion of flies (out of all “trapped” flies) in either of the two traps by treatment. Flies either chose the trap containing pineapple juice adulterated with 10% ethanol (Ethanol + Juice) or pure pineapple juice (Juice). Asterisks denote statistically significant differences by treatment (*P < 0.05).

Given the differences in antennal and OSN activity in flies exposed to IMD (Exp. 2, 3), we asked if these changes in neuron activity affected flies’ performance on an olfactory choice test. We used a modified trap assay^30^ to simultaneously offer two odor traps, one containing pineapple juice (hereafter referred to as “juice”) and one containing pineapple juice adulterated with 10% ethanol by volume (hereafter referred to as “juice + ethanol”). We found significant treatment-level differences in whether flies were found in either of our two odor traps (Fig. 3a, *X*^2^ = 17.11, *P* < 0.001). Only 32% of all flies tested (*N* = 213) were found in either odor trap at the end of the experiment (*N* = 33, 32, and 3 for 0, 10, and 100 µM treatment groups, respectively). Flies treated with 0 and 10 µM were equally likely to become trapped (post-hoc Tukey HSD tests: *P* = 0.21). In contrast, flies treated with 100 µM were significantly less likely to become trapped compared to 0 and 10 µM flies (post-hoc Tukey HSD tests: *P* < 0.001 for both comparisons). Of these trapped flies, 66.7% of the 0 µM flies had a relative preference for juice and 33.3% for juice + ethanol, but this pattern was reversed with exposure to IMD (Fig. 3b). Flies exposed to 10 µM had a significantly higher relative preference for juice + ethanol (59.4% juice + ethanol vs. 40.6% juice) compared to control flies, but this was not true for 100 µM flies (10 µM: *Z-stat* = 2.080, *P* = 0.038, 100 µM: *Z-stat* = 0.012, *P* = 0.990). Of the 3 flies in the 100 µM treatment that were trapped, they were exclusively found in the juice + ethanol trap (0.0% to juice vs. 100% to juice + ethanol).

## Discussion

Using *D. melanogaster* as a model, we examined whether a common neonicotinoid pesticide, imidacloprid (IMD), caused changes in the activity of a single olfactory neuron, whole antenna function, and performance on an olfactory choice test. We found that following exposure to concentrations of IMD that are relevant for fly survival, flies showed a reduction in neuron activity and a delay in return to baseline antennal response. In addition, these effects may scale up to alter olfactory-mediated behavior: flies exposed to IMD had a higher relative preference for pineapple juice containing ethanol than our control group. However, this pattern did not hold for our highest IMD exposure level, as only three flies from this group were found in an odor trap.

Past work has shown that neonicotinoids can disrupt insect sensory systems, including vision and olfaction. In *D. melanogaster,* IMD exposure can damage glial cells and impair vision^31^. In other systems, such as *Locusta migratoria*, IMD exposure has been found to reduce both the peak firing rate of a crucial motion-sensitive neuron and flight avoidance behavior in response to motion-based stimuli^32,33^. In the hoverfly (*Eristalis tenax*), IMD exposure can also reduce stimuli-induced activity in lobula plate tangential cells, which are involved in motion detection in the fly brain^34^. There is also strong evidence to suggest that neonicotinoids affect insect chemoreception, including gustation and olfaction, at least in bees^23,35–37^. Neonicotinoids have been shown to impact specific olfactory regions such as the mushroom bodies and antennal lobes^25–27^, and behaviors such as odor-based learning and navigation^11,22,38^, where their impact may be stronger on olfactory- than visually-guided behavior^11^. For example, a study by Muth et al. demonstrated that bumble bees trained to a specific color-scent discrimination task were more likely to make errors relating to scent identity than color^11^. It is also worth noting that the magnitude of olfactory disruption may vary with taxa and sensory context; studies analogous to ours in moths have shown inconsistent effects (if any) on olfactory neurons involved in pheromone detection^26,38^. In contrast, our results demonstrate that this neonicotinoid pesticide may impair *D. melanogaster* olfactory neurons and olfactory-based behavior.

### Olfactory neuron and antennal activity

Neonicotinoids primarily act as antagonists of nicotinic acetylcholine receptors (nAChRs), which are found throughout the insect central nervous system, and are abundant in the olfactory system, including OSNs^39,40^. Specifically, these pesticides (and IMD, in particular) have been found to target nAChRs subunits Dα1/Dα2/Dβ1/Dβ2^41,42^. In our study, we recorded the activity of an OSN, which expresses the odor receptor Or22a^43^. This neuron expresses the nAChRs subunits Dβ1 and Dβ2 in *D. melanogaster,* although it is probable that one or more alpha subunit is also expressed, but has yet to be identified (Table S1)^44^. When a neonicotinoid binds to these receptors, it initially causes hyperexcitation^45^; however, prolonged exposure to neonicotinoids such as IMD leads to neuron inactivation^40,46^. Our results suggest that prolonged exposure of 100 µM may have induced the inactivation of OSNs, leading to a significantly smaller response to the odor. A recent study by Rabhi et al. found exposure to the neonicotinoid Clothianidin did not alter OSN activity in the black cutworm moth *Agrotis ipsilon*^26^*.* However, these authors fed moths an acute dose of the pesticide, whereas we sustained exposure over 3 days (and focused on a different neonicotinoid). Our approach to pesticide exposure manipulated the concentration of IMD in fly food media. We did not otherwise control the timing of exposure. However, we did confirm that these concentrations of IMD resulted in survival differences (Fig. 1). Both acute and chronic exposure are likely relevant for wild insects, and the differences in the results of these studies may reflect the variation in timing, concentration, or accumulation of exposure.

There may also be system-specific differences in how these chemicals act. Regarding whole antenna response, studies of a one-time topical application of the neonicotinoids Thiacloprid and Clothianidin found that these chemicals can reduce EAG response in one moth species and two bee species (*Lobesia botrana*, *Osmia bicornis*, and *Bombus terrestris*)^38,47^. In contrast, we found *D. melanogaster* showed no difference in the strength of the EAG response with IMD exposure but did show differences in EAG latency to return to baseline response. Although we know neonicotinoids like IMD target nAChRs, it is possible that neonicotinoids also alter neuron function through other mechanisms. For example, these chemicals might change the electrophysiological environment surrounding these neurons or alter neuron function like other neurotoxins shifting the voltage dependence of activation, inhibiting inactivation, or inhibiting ion transport^48^. More studies would be needed to dissect the precise mechanism(s) by which IMD affects EAG response latency.

### Performance on an olfactory choice test

We found that even 10 µM, our lowest exposure of IMD (at which fly survival did not differ significantly from the control, Exp. 1), can change *D. melanogaster’s* relative preference for the presence of ethanol in an odor source. *Drosophila melanogaster* lay their eggs in fermenting fruit and show a preference for alcohol fermentation products such as ethanol^49^. These flies have evolved a tolerance of alcohol and even seek out ethanol when infected by parasites which in turn causes parasite mortality^50^. Whether the relative preference for ethanol in neonicotinoid-stressed flies in our study was driven by self-medication or some other physiological changes remains to be tested. Neonicotinoids also decrease mobility, foraging motivation, and consumption in bees and *D. melanogaster*^21,47,51–54^, which might explain why flies in the 100 µM treatment group had the lowest participation in the test. While our results demonstrate neonicotinoids may alter peripheral odor processing, previous work on antennal lobe and Kenyon cell activity indicate that neonicotinoids may affect olfactory behaviors at multiple levels of information processing^25–27^.

## Conclusion

Neonicotinoid-induced olfactory impairment could affect a diverse assortment of insects, especially pollinators and other insects that depend on olfaction-based navigation and memory much more strongly than sedentary insects like aphids^28,55^. Utilizing the extensively annotated olfactory system of *D. melanogaster* (which is well-conserved among insects) allows us to understand the neurological basis of neonicotinoids’ behavioral effects at a higher degree of resolution than is possible in other insect systems. Such studies might unmask the mechanism by which these compounds alter neuron function, which in turn affects important behaviors such as foraging or mating that might be missed by traditional assays. By evaluating the subtle effects of such pesticides in model systems like *D. melanogaster*, we can continue to understand the scope of their impact and role in the decline of beneficial insect populations such as pollinators.

## Methods

We maintained Canton-S WT of *Drosophila melanogaster* on standard cornmeal-dextrose agar (prepared according to manufacturer’s protocol: Genesee Scientific, San Diego, California) on a 12-h light cycle at 25 °C and 60% relative humidity, unless otherwise specified. Upon eclosure, we transferred adult flies to experimental media (see below). 

### Chemical reagents

To create a 1:1 stock solution, we dissolved IMD (Sigma-Aldrich, USA) in dimethyl sulfoxide (DMSO), which was then pipetted into the food during preparation at concentrations ranging from 0, 10, 50, and 100 µM. We supplemented food media with indigo-carmine dye (Sigma-Aldrich, USA) to visually confirm food consumption.

### Experiment 1: Fly survival

To confirm our IMD exposure protocol, we performed a separate survival assay to confirm the exposure protocol (Fig. 1). Following the protocol established by Daisley et al.^29^, we transferred newly eclosed adult flies to media (prepared as above) containing 0, 10, 50, and 100 µM of IMD. Fly survival was monitored daily for 15 days.

### Experiment 2: Single neuron function

We performed single-unit olfactory sensory neuron (OSN) recordings on flies (*N* = 14) following^56^. Newly eclosed flies were exposed to diets prepared as described above to contain 0, 10, and 100 µM of IMD for three days. All recordings were conducted as previously described^43,57^. Briefly, we recorded neuron activity from the AB3 sensilla immediately following stimulation with methyl hexanoate (Fig. S1). Methyl hexanoate is a compound found in ripe fruit (such as pineapples, passion fruit, and strawberries). It is known to elicit a strong response in OSNs^58^. We presented odor stimuli using a Pasteur pipette containing 50 µL of methyl hexanoate diluted in paraffin oil (10^−4^ dilution) placed on a Whatman 13-mm filter paper disk (Millipore). We prepared these odor cartridges shortly before odor presentation, and each cartridge was never used more than three times. Three recordings were taken from three separate AB3 sensilla for each individual where possible (*N* = 20, 30, and 22 recordings per treatment, respectively). Then, we assessed the spike frequency of the AB3a neuron in the first 500 ms post-stimulus. The AB3a neuron expresses the odor receptor Or22a, which is sensitive to methyl hexanoate^43^. The mean response frequency to DMSO alone (which includes spontaneous response) was subtracted from each odor response frequency for each recording.

### Experiment 3: Antennal response

We performed whole antenna electroantennograms (EAG) following^59,60^ on newly eclosed flies which had been exposed to diets containing either 0 or 100 µM of IMD (*N* = 5 and 4 individuals, respectively) for three days. We placed the recording electrode in the antennal region rich in AB3 sensilla and conducted three recordings per individual (*N* = 14 and 10 recordings, respectively) immediately following stimulation with methyl hexanoate diluted in paraffin oil (10^−4^ dilution, preparing odor cartridges as above). We quantified antennal response as the minimum, the integral of the response curve, and the time to return to baseline (0 mV) for each recording.

### Experiment 4: Performance on an olfactory choice test

To assess the potential impacts of IMD on olfactory-guided behavior, we used a modified trap assay^30^ where flies were simultaneously offered two odorants representing ripe vs. unripe fruit. Assays took place inside experimental chambers (dimensions 24.75 × 14.5 × 11.5 cm) housed in an incubator on a 24-h dark cycle to reduce the influence of visual stimuli. Odor traps were constructed from clear plastic 12-dram vials. Each odor trap was fitted with the cut end of a 1000 µL pipette tip, allowing flies to enter the trap, but not exit.

One odor trap contained 1000 µL unadulterated canned liquid pineapple juice (Dole Food Company and Total Produce, USA, which was kept frozen until use). The other contained pineapple juice adulterated with 10% ethanol by volume. Ethanol, a byproduct of the fermentation process, should be attractive to mated female fruit flies, which lay eggs in fermenting fruit^49^. In addition, ethanol has been shown to elicit a strong EAG response in *D. melanogaster,* and when used in a blend with other compounds commonly found in fermenting fruit, the blend lured and trapped more flies than compounds in isolation^61^. Flies were exposed to IMD as in experiments 2 and 3, but in this experiment, 10 female and 10 male flies were placed in each vial to allow mating. After 3 days of exposure, adult females were transferred to an empty vial where they were starved (given no food or water) to increase participation in the assay. After 24 h, flies were then transferred to an experimental chamber. Within a chamber, all flies were assigned to the same treatment (0, 10, or 100 µM). After 48 h, we counted flies in each odor trap and the number of untrapped flies remaining in the experimental chamber.

### Analyses

All statistical analyses were carried out in R version 4.0.2^62^. To identify differences in fly survival by treatment, we performed pairwise comparisons of survival by treatment using a Log-Rank test from the survival package^63^. To compare OSN response by treatment, we used a linear mixed-model approach treating individual as a random effect using the lme4 package^64^ and type II ANOVA using the ANOVA function from the car package^65^ and Tukey comparison was performed using the glht function from the multcomp package^66^. Electroantennogram response analysis again used a linear mixed-model approach treating individual as a random effect, but here we used three separate models for each of our three response variables: minimum, integral, and return to baseline for each treatment. To assess performance on an olfactory test, here we used a binomial linear regression to compare the total number of flies in either odor trap (“trapped flies”) to flies in the larger experimental chamber (“untrapped flies”) for each treatment using the stats package^67^ and type II ANOVA using the ANOVA function from the car package^65^ and Tukey comparison was performed using the glht function from the multcomp package^66^ as before. This allowed us to estimate the effects of the insecticide on fly movement and/or feeding motivation, two aspects of behavior for which IMD has well-documented effects^18^. Finally, to assess if IMD also alters odor discrimination, we used a separate binomial regression to compare the number of flies in the unadulterated juice odor trap and the adulterated (juice + 10% ethanol) juice odor trap.

## Supplementary Information

Supplementary Information.

## Data Availability

The datasets and R scripts for statistical analysis generated by this study are available on request to the corresponding author.
