# Supplementary material for: A neonicotinoid pesticide alters Drosophila olfactory processing
Source: Sci Rep. 2023 Jun 30;13:10606. doi: 10.1038/s41598-023-37589-w (PMC10313779; doi:10.1038/s41598-023-37589-w)
Supplement: Supplementary file 1 — Supplementary Information. [file 41598_2023_37589_MOESM1_ESM.docx]

**A neonicotinoid pesticide alters *Drosophila* olfactory processing**

Anna R. Tatarko*, Anne S. Leonard, Dennis Mathew

**Supplementary Material**


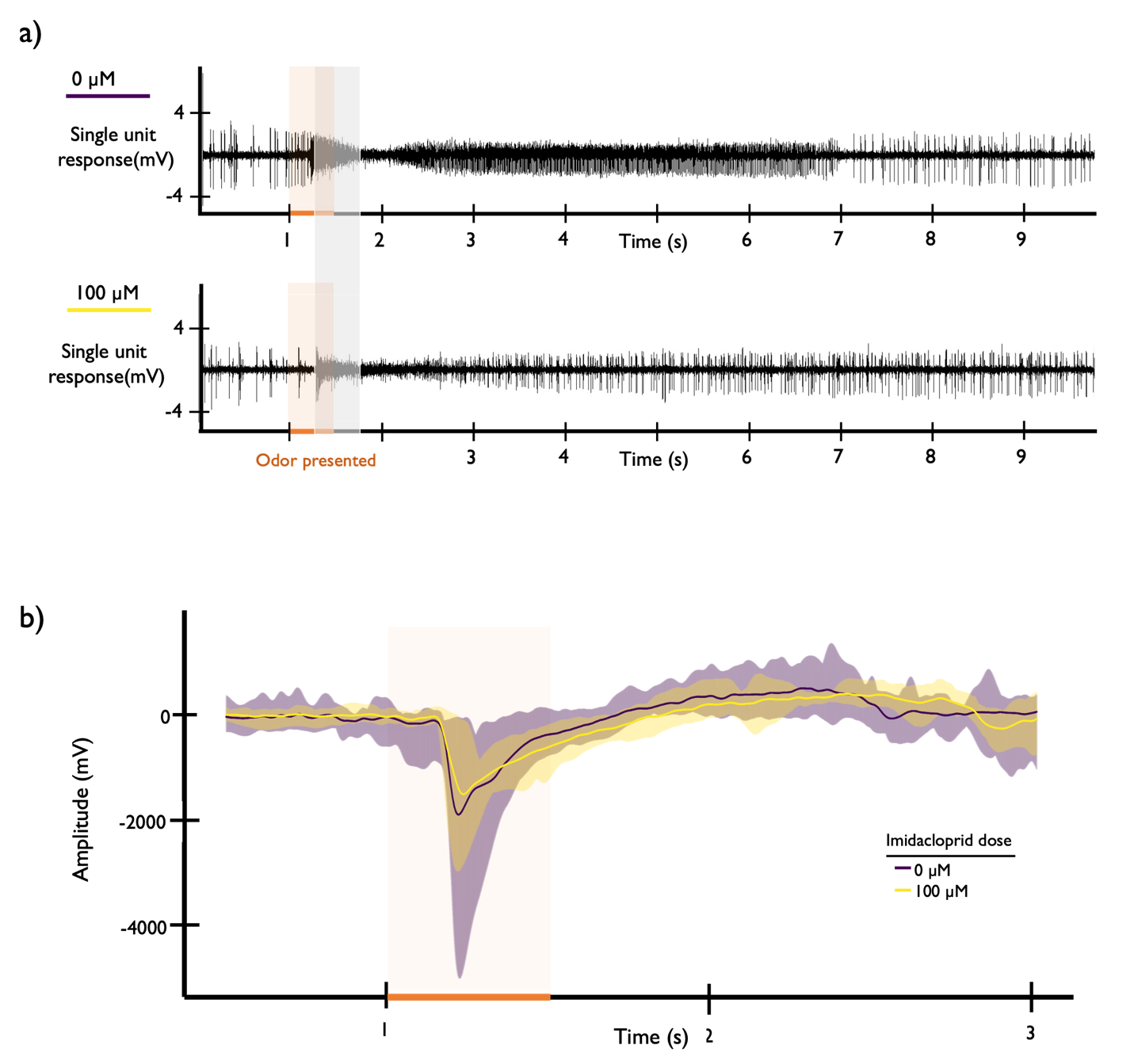


Fig S1. Examples of the AB3 neuron’s activity in response to odor stimulus in Exp. 2 (odor presented at T = 1 second, denoted with an orange box) over ten seconds (a). We quantified the response in the first 500 milliseconds of odor detection (denoted with a shaded gray box). Responses are shown in a fly dosed with 0 µM (top) of imidacloprid and a fly dosed with 100 µM (bottom) of IMD. (b) EAG response to an odor in response to an odor measured in Exp. 3 (odor presented at T = 1 second, denoted with an orange box). Flies were dosed with 0 µm (with individual response in a purple cloud with mean response in bold) or 100 µM IMD (with individual response in a yellow cloud with mean response in bold).

Table S1. Expression of the Dα1, Dα2, Dβ1, and Dβ2 subunits of antennal nicotinic acetylcholine receptors (nAChRs) by cell type examined. Specifically, our single-unit recordings were from the olfactory receptor neuron Or22a which we have bolded and highlighted here. Data were compiled from Fly Cell Atlas online interactive Automated Single-Cell Analysis Pipeline “ASAP” (see reference 43).

|  | **Subunit of nAChR Expressed** | | | |
| --- | --- | --- | --- | --- |
| **Cell Type Examined** | **Dα1** | **Dα2** | **Dβ1** | **Dβ2** |
| Adult Antennal Glial Cell | X |  | X | X |
| Adult Glial Cell |  |  |  |  |
| Adult Olfactory Receptor Neuron Gr21a/63a | X |  | X | X |
| Adult Olfactory Receptor Neuron Ir56a+, Orco- |  |  |  | X |
| Adult Olfactory Receptor Neuron Ir75d | X |  | X | X |
| Adult Olfactory Receptor Neuron Ir84a, Ir31a, Ir76a, Ir76b, Ir8a, Or35a | X |  |  | X |
| Adult Olfactory Receptor Neuron Or13a |  |  |  | X |
| **Adult Olfactory Receptor Neuron Or22a, Or42b, Or59b** |  |  | **X** | **X** |
| Adult Olfactory Receptor Neuron Or47a, Or56a and likely other ORN types | X |  | X |  |
| Adult Olfactory Receptor Neuron Or47b |  |  | X | X |
| Adult Olfactory Receptor Neuron Or65 |  |  | X |  |
| Adult Olfactory Receptor Neuron Or67a and likely other unknown ORN types | X |  | X | X |
| Adult Olfactory Receptor Neuron Or67d |  |  | X | X |
| Adult Olfactory Receptor Neuron Or83c, Or82a |  |  | X |  |
| Adult Olfactory Receptor Neuron Or85a, Or43b |  |  |  |  |
| Adult Olfactory Receptor Neuron Or88a |  |  |  | X |
| Adult Olfactory Receptor Neuron Or92a | X |  | X |  |
| Adult Olfactory Receptor Neuron unkown type Orco- |  |  |  |  |
| Adult Olfactory Receptor Neuron unkown type Orco+ | X |  | X | X |
| Adult Olfactory Receptor Neuron, Acid-Sensing Ir64a |  |  |  |  |
| Adult Olfactory Receptor Neuron, Acid-Sensing Ir75a/b/c, Ir64a | X |  |  | X |
| Arista and Sacculus Thermosensory and Hygrosensory Neuron Ir21a, Ir40a, Gr28b | X |  |  |  |
| Epithelial Cell | X |  | X | X |
| Hemocyte |  |  |  |  |
| Johnston Organ Neuron | X | X |  | X |
| Muscle Cell |  |  | X | X |
| Olfactory Receptor Neuron, Coeloconics |  |  |  |  |
| Sacculus/Arista Neuron | X |  |  | X |
| Unannotated | X | X | X | X |
